# DEVELOPING INTEREST IN GEROPSYCHOLOGY THROUGH INTERGENERATIONAL EXPERIENCE

**DOI:** 10.1093/geroni/igz038.2228

**Published:** 2019-11-08

**Authors:** Katherine King, Erlene Rosowsky, Michelle Jolson

**Affiliations:** William James College, Newton, Massachusetts, United States

## Abstract

In order to develop interest in geropsychology, it is beneficial for students to have direct experiences with older adults. This presentation reports on the development of an innovative student volunteer group within a doctoral program in clinical psychology. In this group, students engage directly with older adults in the community. With the help of geropsychology faculty, students have developed and led intergenerational activities such as a panel discussion about dating, reminiscence using music, fraud recognition, and social media training. The group consists of 32 current students, along with 7 recent graduates. Community partners have provided overwhelmingly positive feedback. Students have reported benefits including personal satisfaction from intergenerational contact, learning and pleasure from hearing older adults’ stories, and feeling appreciated. Reported challenges include communication issues and feeling unsure how to relate to people with dementia. Students also describe negative assumptions about aging that have changed as a result of their participation.

